# Supplementary material for: The genome sequence of the Large Red Tailed Bumble Bee, Bombus lapidarius (Linnaeus, 1758)
Source: Wellcome Open Res. 2025 Feb 12;10:67. [Version 1] doi: 10.12688/wellcomeopenres.23624.1 (PMC11971630; doi:10.12688/wellcomeopenres.23624.1)
Supplement: Peer review history

# Reviewer report: Reviewer response for version 1

**DOI:** 10.21956/wellcomeopenres.26064.r119100

**Published:** 2025-04-04

**Authors:** Eduardo E Zattara

**Affiliations:** 1 Instituto de Investigaciones en Biodiversidad y Medioambiente, CONICET-Universidad Nacional del Comahue, Bariloche, Argentina

This data note presents the sequencing and assembly of the genome of the bumblebee *Bombus lapidarius,* in the context of the Darwin Tree of Life initiative. Using state of the art techniques, the assembly includes 18 scaffolds (the typical haploid chromosome count for the genus) that comprise 81% of the assembled sequence. Although the results from BlobTools suggest that a significant number of scaffolds represent sequence from genomes other than that of the target species, there is no comment about the almost 20% of assembled sequence remaining outside of those 18 scaffolds. However, given the context of this study, it is likely that such analyses, along with comparisons to other genomes, are forthcoming.

 The one thing that is missing is making explicit the sex of the sequenced individual, which considering the haplodiploid nature of Hymenoptera, is a meaningful point.

Are sufficient details of methods and materials provided to allow replication by others?

Yes

Is the rationale for creating the dataset(s) clearly described?

Yes

Are the datasets clearly presented in a useable and accessible format?

Yes

Are the protocols appropriate and is the work technically sound?

Yes

Reviewer Expertise:

Conservation, Genomics, Transcriptomics

I confirm that I have read this submission and believe that I have an appropriate level of expertise to confirm that it is of an acceptable scientific standard.

# Reviewer report: Reviewer response for version 1

**DOI:** 10.21956/wellcomeopenres.26064.r119093

**Published:** 2025-02-25

**Authors:** Christopher Wyatt

**Affiliations:** 1 University College London, London, England, UK

The authors have provided a high quality chromosome-level genome for *Bombus lapidarius*, as shown in the high N50 statistics and genome completeness of BUSCO genes. 

 The rational was explained clearly, along with a highly detailed methods of the work. It is a great addition, with many Bombus genomes now available through Darwin Tree of Life.

 As mentioned in a prior review of wasp genomes, the mitochondrial genome seems too big for a Bombus species. The mitochondrial genomes ( **mitogenomes**) of **Bombus** (bumblebees) typically range in length from **15,700 to 18,000 base pairs (bp), **so this is way bigger, and not sure if there is an explanation for this.

Are sufficient details of methods and materials provided to allow replication by others?

Yes

Is the rationale for creating the dataset(s) clearly described?

Yes

Are the datasets clearly presented in a useable and accessible format?

Yes

Are the protocols appropriate and is the work technically sound?

Yes

Reviewer Expertise:

genomics, transcriptomics, evolutionary biology

I confirm that I have read this submission and believe that I have an appropriate level of expertise to confirm that it is of an acceptable scientific standard.
